# Cortisol Impacted on Explicit Learning Encoding, but Not on Storage and Retrieval, and Was Not Associated With Sleep Patterns—Results From the Trier Social Stress Test for Children (TSST-C) Among 9-Years Old Children

**DOI:** 10.3389/fpsyg.2018.02240

**Published:** 2018-11-21

**Authors:** Serge Brand, Thorsten Mikoteit, Nadeem Kalak, Dena Sadeghi Bahmani, Sakari Lemola, Markus Gerber, Sebastian Ludyga, Madleina Bossard, Uwe Pühse, Edith Holsboer-Trachsler, Martin Hatzinger

**Affiliations:** ^1^Center for Affective, Stress and Sleep Disorders (ZASS), University of Basel, Psychiatric Clinics (UPK), Basel, Switzerland; ^2^Division of Sport Science and Psychosocial Health, Department of Sport, Exercise and Health, University of Basel, Basel, Switzerland; ^3^Substance Abuse Prevention Research Center, Kermanshah University of Medical Sciences, Kermanshah, Iran; ^4^Sleep Disorders Research Center, Kermanshah University of Medical Sciences, Kermanshah, Iran; ^5^Psychiatric Services Solothurn, Solothurn, Switzerland; ^6^Psychiatric Clinics (UPK), University of Basel, Basel, Switzerland; ^7^Max Planck Institute of Psychiatry, Munich, Germany; ^8^Isfahan Neurosciences Research Center, Alzahra Research Institute, Isfahan University of Medical Sciences, Isfahan, Iran; ^9^Department of Psychology, University of Warwick, Coventry, United Kingdom

**Keywords:** Trier Social Stress Test for Children, learning, sleep, cortisol secretion, figural learning, bidirectionality, 9 years-old children

## Abstract

**Background:** Learning is the relatively permanent change of behavior as a result of experience and tightly related to memory and cognition. Learning is particularly important for children. Further, restoring sleep is associated both with improved learning performance and lower cortisol levels as a proxy of the so-called hypothalamus-pituitary-adrenocortical axis activity (HPA-AA). With the present study, we investigated, if and to what extent explicit learning performance was associated with cortisol levels at baseline and under challenge conditions and with objective sleep dimensions among 9-years old children.

**Methods:** A total of 39 children (mean age = 9.5 years; 39% females) took part in the study. Verbal and figural working and long-term memory were tested before and after the Trier Social Stress Test for Children (TSST-C). Further, children underwent sleep-EEG assessment, and cortisol awakening response (CAR) was assessed.

**Results:** Higher cortisol levels were associated with lower explicit learning encoding (verbal, but not figural learning). Higher verbal and figural working and long-term memory performance predicted lower cortisol secretion under the TSST-C, along with higher verbal and figural working and long-term memory performance after the TSST-C. Cognitive test performances were not mediated by cortisol secretion under the TSST-C. Cognitive performance, cortisol secretion under challenge (TSST-C) and basal conditions (morning) and sleep patterns were unrelated.

**Conclusions:** The pattern of results suggests that among a sample of 9-years old children cortisol secretion and stages of memory processes (encoding, storage, retrieval) are associated in a complex and bi-directional way. Further, it appears that cognitive-emotional processes underlying cognitive performance and its evaluation might impact on subsequent cortisol secretion as a proxy of neuroendocrinological response to cognitive-emotional processes. Last, cognitive performance and cortisol secretion under challenge conditions were not related to objective sleep patterns and baseline cortisol secretion.

## Introduction

Learning is the relatively permanent change in behavior as a result of own experiences. In humans, complex learning is associated with a broad range of cognitive and emotional processes, which are reflected by neurophysiological changes in synaptic plasticity. Typical steps of learning are encoding, storage, and retrieval of information to and from long-term memory. Further, already Hebb (1955) underlined the importance of arousal for learning, where physiologically, arousal refers to the level of alertness of an organism; psychologically, arousal refers to the tension, ranging from calmness to anxiety. Specifically, Hebb's concept of arousal based on the concept of Yerkes and Dodson (1908), which related arousal to performance. Briefly, the Yerkes and Dodson law claims that (cognitive, behavioral) performance is best at an intermediate level of physiological arousal: while a too low level of arousal (e.g., feeling of being bored; illness; somnolence; fatigue; lack of interest) is related to low performance, a too high level of arousal (e.g., anxiety, panic, state of mania) is also related to poor performance. In this view, above all the state of anxiety and panic is tightly related to an overactivated organism, and such over-activation is neurophysiologically reflected by an increased hypothalamus-pituitary-adrenocortical axis activity (HPA-AA; Miller et al., 2007; Holsboer and Ising, 2010) with cortisol as the ultimate indicator variable.

In the meanwhile, several meta-analyses showed that poor learning performance and increased cortisol levels were associated (Sauro et al., 2003; Het et al., 2005; Shields et al., 2015, 2016, 2017; Starcke and Brand, 2016). The underlying concept claims that acute stress, understood as the occurrence of a transient stressor, triggers a cascade of physiologically adaptive changes to cope with the stressor. Among others, a stressor increases the activation of the sympathetic-adrenal-medullary (SAM) axis, the hypothalamic-pituitary-adrenal axis (HPA-A) with the subsequent release of adrenal hormones such as cortisol, and upregulation of the immune system and inflammatory activity (Allen et al., 2014). Such physiological adaptations to cope with the stressor have adverse consequences on cognitive processes, specifically on executive functions (i.e., top-down guidance of behavior toward a specific goal) such as working memory, cognitive flexibility and inhibition (Shields et al., 2016, 2017). To explain such an impairment on cognitive functions, it is claimed that cortisol spikes interrupt typical prefrontal cortical function (Porcelli et al., 2008; Vogel et al., 2016). Henckens et al. (2011, 2012) showed that elevated cortisol levels impaired both working memory and inhibition. Further, blocking cortisol receptors decreased the impairing impact of acute stress on executive functions (Schwabe et al., 2013).

Next, while there is extant evidence of such association between stress, cortisol release and impaired executive functions among adults (Sauro et al., 2003; Het et al., 2005; Shields et al., 2015, 2016, 2017; Starcke and Brand, 2016), surprisingly, research on such associations among children is scarce. de Veld et al. (2014) assessed 158 children (mean age: 10.6 years). First, children performed a working memory and delayed retrieval test under control conditions; 1 week later, they repeated the tests under stress conditions, that is: retrieval occurred during the session of psychosocial stress (Trier Social Stress Test for Children (TSST-C; Buske-Kirschbaum et al., 1997). Outcome variables were the performance of cognitive tests and the levels of salivary alpha-amylase and saliva cortisol. Results showed that correlations between physiological stress responses and cognitive performance were generally low, that is to say: Alpha-amylase levels and cortisol levels were only modestly associated. Further, de Veld et al. (2014) observed an inverted U-shape association between very low and very high cortisol secretions and poor performance, confirming therefore the law of Yerkes and Dodson (1908) that optimal, but not maximal arousal predicted highest performance. Quesada et al. (2012) assessed 44 children aged 8–10 years, who were randomly assigned either to the control or to the intervention condition. Intervention condition consisted of the TSST-C (Buske-Kirschbaum et al., 1997; see also below). Results showed that compared to children in the control condition, children in the intervention condition had higher cortisol levels, lower mood and a poorer performance in the delayed memory task, while no group differences were found for the working memory performance.

Collectively, research on the association of increased stress, higher cortisol secretion and poorer executive functions is abundant among adults (Sauro et al., 2003; Het et al., 2005; Shields et al., 2015, 2016, 2017; Starcke and Brand, 2016), whereas research on the same research question among children is scarce (Quesada et al., 2012; de Veld et al., 2014). Accordingly, the first aim of the present study was to investigate, if and if so to what extent psychosocial stress (here: TSST-C) might impact on delayed working memory retrieval among 9-years old children. Second, previous studies (Quesada et al., 2012; de Veld et al., 2014) have focused on verbal learning performances. However, there is extant evidence to show that lexical and graphem information (Sternberg, 2017) are stored as distinguished cognitive information processes. Accordingly, the second aim of the present study was to assess, if and to what extent retrieval of graphem information might be influenced by psychosocial stress (here: TSST-C). To do so, children completed the Rey Osterrieth Complex Figure test (Meyers, 1995).

Last, there is evidence that poor sleep and increased morning cortisol awakening response (CAR) are associated both among children (Hatzinger et al., 2008, 2010, 2012, 2013b, 2014) and adults (Hori et al., 2011; Minkel et al., 2014; van Dalfsen and Markus, 2018). Further, cortisol secretion under basal conditions (that is, CAR) and under stress conditions appeared to be related (Hatzinger et al., 2007; Lemola et al., 2015; Perkinson-Gloor et al., 2015; Maurer et al., 2016). Accordingly, the third aim of the present study was to investigate the associations between sleep patterns and cortisol secretion under baseline (morning cortisol) and challenge (TSST-C) conditions.

The following four hypotheses and one research question were formulated. First, following others (Sauro et al., 2003; Het et al., 2005; Quesada et al., 2012; de Veld et al., 2014; Shields et al., 2015, 2016, 2017; Starcke and Brand, 2016; van Dalfsen and Markus, 2018), we assumed that higher cortisol levels would be negatively associated with memory encoding. Second, we expected that a psychosocial stressor impaired retrieval of verbal working memory. Third, following others (Hatzinger et al., 2008, 2010, 2012; Hori et al., 2011; van Dalfsen and Markus, 2018), we assumed that poor sleep and increased morning cortisol secretion were associated. Fourth, following others (Hatzinger et al., 2007; Lemola et al., 2015; Perkinson-Gloor et al., 2015; Maurer et al., 2016), we expected that poor sleep, and cortisol level under baseline (morning cortisol) and under challenge conditions (TSS-C) would be associated. Finally, we examined as exploratory research question, if and to what extent retrieval of working memory performance of figural information (Rey Osterrieth Complex Figure Test) would be associated with cortisol levels as a proxy of psychosocial stress (TSST-C).

To test our hypotheses and to examine our research questions, a sample of 39 children aged about 9 years were assessed in their cognitive performance and cortisol levels before and after a psychosocial stress, along with the objective sleep and cortisol levels in the morning. We had two reasons to assess 9 years old children: First, Hollanders et al. (2017) showed in their systematic review on the cortisol secretion under the TSST-C that mainly children and adolescents at the age of 8, and 10 to 16 years were assessed, while research on 9 years old children was less frequent. Second, the present sample belongs to a larger sample of a long-term study on the associations between psychological functioning, sleep and cortisol secretion; children were assessed at the age of 5, 6, 9, and 14 years, and here, we present data of children at the age of 9 years. Here, children underwent a slightly modified and age-adapted TSST-C protocol, while in previous studies at the age of 5 and 6 years, for want of an appropriate TSST-C, children underwent the MacArthur Story Stem Battery (Hatzinger et al., 2007, 2008, 2010).

## Methods

### Procedure

Children from a larger long-term study on sleep, behavior and the HPA axis activity among pre-schoolers and children participated in this study (Hatzinger et al., 2007, 2008, 2010, 2012, 2013a,b, 2014; Mikoteit et al., 2012, 2013; Brand et al., 2015; Sadeghi Bahmani et al., 2016). At children's age of about 9 years, children and their parents were informed about the aims of part of the present study. They were assured about anonymous data gathering; then, both children and parents signed the written informed consent. Next, children underwent an in-home sleep-EEG assessment, and morning saliva samples were gathered. In the afternoon of the subsequent day, children underwent the Trier Social Stress Test for Children (TSST-C). Before and after the TSST-C children underwent cognitive testing, while seven saliva samples were gathered from the beginning till the end of the entire testing. The local ethical committee approved the study (Ethik Kommission Basel, Basel, Switzerland), which was performed in accordance of the rules laid down at the Declaration of Helsinki and its later amendments.

While the present results were a part of a larger ongoing study (see above), data were not published in previous papers; thus, the present results are novel.

### Sample

A total of 39 children (mean age = 9.5 years, SD = 0.39; 38.4% females) took part in the present study. A brief medical and psychiatric examination (brief interview with parents; medical records and previous study records) ensured that exclusively typically developing and currently somatically and psychologically healthy children took part in the study. Inclusion criteria were: 1. Already participant of the ongoing study (Hatzinger et al., 2007, 2008, 2013a, 2014); 2. willing and able to comply with the study conditions; 3. parents' and children's signed written informed consent. Exclusion criteria were: 1. Current health issues such as flu, injury, psychological or psychiatric issues or similar; 2. intake of mood-, attention- or sleep-altering substances; 3. known skin allergy to electrodes or patches.

### Cognitive testing

Two tests were employed to assess verbal and figural working memory and long-term memory.

For verbal working memory and long-term memory, the Rey Auditory Verbal Learning Test (Schmidt, 1996) was employed. For working memory, a list of 15 words was read, and the child had to reproduce as many words as possible (immediate and free recall; word order is not important). This trial was repeated three times. Next, a word list of 30 words was presented, and the child had to recognize the correct words of the presented list (immediate and cued recall; again, the word order was not important). The delayed free and cued recall was performed after the TSST-C. The outcome measurement is the number of correctly recalled items.

For figural working memory and long-term memory, the Rey Osterrieth Complex Figure Test (Meyers, 1995) was employed. For working memory, a complex figure was presented, and the child had to reproduce the figure as detailed as possible (immediate and free recall). The delayed free recall was performed after the TSST-C. The outcome measurement is the accuracy and the number of correctly drawn elements of the figure.

### Sleep-EEG assessment

The in-home sleep-EEG assessment has been extensively described in Hatzinger et al. (Hatzinger et al., 2008, 2012, 2013b; Brand et al., 2015). Children's sleep was recorded with an ambulatory EEG system (Oxford-Medilog 9200; Oxford Medical Diagnostics, Oxford UK). As a standard procedure, four electrodes were placed at C3-A2 and C4-A1 (EEG), two electrodes were placed on the chin for electromyogram (EMG) and two electrodes on the right and left side to register the electrooculogram (EOG). A1 and A2 electrodes were physically linked. As this assessment was children's fourth sleep-EEG assessment (one mock assessment and one full assessment at the age of 5 years, and at the age of 6 years (Hatzinger et al., 2008, 2012, 2013b), a further assessment to avoid any “first night effect” was not necessary anymore. Sleep EEGs were visually analyzed by two qualified raters according to the standard procedures proposed by Rechtschaffen and Kales (Anderer et al., 2007) (inter-rater reliability: kappa = 0.94).

### HPA system activity under baseline conditions (CAR: cortisol awakening response)

Stalder et al. (2016) have shown that the morning cortisol response with strict reference to the time of awakening is a reliable index of basal HPA axis activity, irrespective from time of awakening, sleep duration, physical activity, or morning routines. As described elsewhere (Hatzinger et al., 2008, 2012; Clow et al., 2010; Stalder et al., 2016), and given that children repeatedly participated in the saliva sampling method during the ongoing study, parents just supervised how children took four saliva cortisol samplings in the morning at 0, 10, 20, and 30 min after awakening. Waking time ranged from 6.30 to 7.15 a.m.

### HPA system activity under non-pharmacological challenge conditions

The Trier Social Stress Test for Children (TSST-C Buske-Kirschbaum et al., 1997) is a well-established non-pharmacological test to challenge the HPA-axis activity (Gunnar et al., 2009). Briefly, children had to accomplish a thrilling story and to present the end of this story in front of a (mock) jury within an exact time period of 4 min. Further, they were told to speak loudly and clearly in the (mock) microphone, while the entire session was registered on a (mock) camera. No feedback was given, and children were stopped when trespassing the 4-min time frame, or children were told to continue the story, in case they stopped before the 4 min. The next task consisted of mental calculation: Children had to count down loudly from 758 minus 7, and to restart in case of a mistake. As described elsewhere (Buske-Kirschbaum et al., 1997; Gunnar et al., 2009; Stadelmann et al., 2018), the set-up of the TSST-C is such to induce (social) anxiety and uncontrollability, which, by definition, trigger psychosocial stress, which is neuroendocrinologically measured by saliva cortisol samplings. Seven saliva samples were taken as described in Table 1.

**Table 1.1:** Correlations (Pearson's Correlation) between the TOTAL AUC_CAR_ (cortisol awakening response), AUC_ANTE_, AUC_INDUCTION_, and AUC_POST_.

	**AUC_CAR_**	**AUC_ANTE_**	**AUC_INDUCTION_**	**AUC_POST_**
	**M (SD)**	**M (SD)**	**M (SD)**	**M (SD)**
	**395.04 (167.75)**	**47.88 (21.01)**	**12.62 (4.93)**	**49.29 (16.92)**
AUC_CAR_	–	−0.024	0.45^**^	−0.082
AUC_ANTE_		-	−0.17	−0.20
AUC_INDUCTION_			–	0.09
AUC_POST_				–

CAR, cortisol awakening response; AUC, area under the curve; AUC_ANTE_, AUC before stress induction; AUC_INDUCTION_, AUC during stress induction; AUC_POST_, AUC after stress induction.

** *p < 0.01*.

### Saliva cortisol sampling technique and cortisol analysis

As described elsewhere (Hatzinger et al., 2008, 2013a), saliva samples were obtained using the “Salivette” device for quick and hygienic sampling (Sarstedt, Nümbrecht/Germany). This device includes a small cotton swab, on which the subject gently chews for about one min. Thereafter, the swab is transferred into a small plastic tube, the Salivette container, and stored in the freezer. For morning cortisol measurements, saliva sampling was started immediately after awakening without first rinsing the mouth with water. In order to avoid contamination of saliva with food or drinks or with blood caused by micro-injuries in the oral cavity, participants were asked not to eat breakfast or to brush their teeth before sampling was completed. Saliva samples were returned to the laboratory, where samples were centrifuged at 4°C (2,000 rpm, 10 min) and stored at −20°C until assay. Next, free salivary cortisol concentrations were analyzed using a time-resolved immunoassay with fluorometric detection “Coat-A- Count” Cortisol RIA from DPC (Diagnostics Products Corporation; obtained trough H. Biermann GmbH, Bad Nauheim, Germany). Intra- and inter-assay variabilities of this assay were < 2.12 and 2.98%, respectively.

### Statistical analysis

For cortisol secretion, the area-under-the-curve (AUC_G_) was calculated, using the trapezoidal integration. The baseline cortisol secretion (morning cortisol/CAR; Cortisol Awakening Response) was labeled as AUC_CAR_. The AUC between the beginning of the examination (see Table 1; Introduction and briefing) and the TSST-C was labeled AUC_ANTE._ The AUC under the TSST-C condition was labeled AUC_INDUCTION_. The AUC from the end of the TSST-C to the debriefing was labeled AUC_POST._

**Table 1.2:** Correlations (Pearson's Correlation) between the BASAL AUC_CAR_ (cortisol awakening response), AUC_ANTE_, AUC_INDUCTION_, and AUC_POST_.

	**AUC_CAR_**	**AUC_ANTE_**	**AUC_INDUCTION_**	**AUC_POST_**
	**M (SD)**	**M (SD)**	**M (SD)**	**M (SD)**
	**211.55 (114.53)**	**38.70 (19.45)**	**11.06 (5.56)**	**38.70 (19.45)**
AUC_CAR_	–	−1.49	0.50	0.50
AUC_ANTE_		–	−0.26	−0.26
AUC_INDUCTION_			–	0.90^***^
AUC_POST_				–

CAR, cortisol awakening response; AUC, area under the curve; AUC_ANTE_, AUC before stress induction; AUC_INDUCTION_, AUC during stress induction; AUC_POST_, AUC after stress induction.

*** *p < 0.001*.

To compare the cognitive performances before and after the stress challenge, two *t*-tests for paired samples (verbal; figural) were performed.

A series of Pearson's correlations were performed to calculate the association between AUCs and cognitive performances (immediate retrieval; delayed retrieval; verbal and figural learning). A series of partial correlations were performed between the AUC_INDUCTION_ and cognitive performance after the stress induction (delayed retrieval), controlling for the cognitive performance before the stress induction.

A series of Pearson's correlations were performed to calculate the associations between cortisol secretions under baseline and challenge conditions, sleep parameters, and cognitive performance.

The level of significance was set at alpha ≤ 0.05. All statistical computations were performed with SPSS® 25.0 (IBM Corporation, Armonk NY, USA) for Apple® Mac®.

## Results

### Verbal and figural performance before and after stress induction

Verbal performance (free and cued recall) decreased from immediate recall [*M* = 10.08, *SD* = 1.09) to delayed recall (*M* = 7.85, *SD* = 1.45; *t*_(38)_ = 9.72, *p* = 0.001, *d* = 1.76].

Figural performance decreased from immediate recall (*M* = 18.33, *SD* = 5.38) to delayed recall [*M* = 17.75, *SD* = 4.31; *t*_(38)_ = 4.00, *p* = 0.001, *d* = 0.14].

### Cortisol at baseline (CAR), and under challenge conditions (AUC_ANTE_, AUC_INDUCTION_, AUC_POST_)

Table 1 (Tables 1.1–1.3) shows the descriptive and correlational statistical indices of cortisol secretions (AUC_CAR_, AUC_ANTE_, AUC_INDUCTION_, AUC_POST_). Higher TOTAL AUC_CAR_ levels were associated with higher AUC_INDUCTION_ levels (*r* = 0.45, *p* = 0.004). All other correlation coefficients were not significant and therefore trivial. Higher BASAL AUC_INDUCTION_ levels were associated with higher BASAL AUC_POST_ levels. Higher NETTO AUC_ANTE_ levels were associated with lower NETTO AUC_INDUCTION_ levels.

**Table 1.3:** Correlations (Pearson's Correlation) between the NETTO AUC_CAR_ (cortisol awakening response), AUC_ANTE_, AUC_INDUCTION_, and AUC_POST_.

	**AUC_CAR_**	**AUC_ANTE_**	**AUC_INDUCTION_**	**AUC_POST_**
	**M (SD)**	**M (SD)**	**M (SD)**	**M (SD)**
	**183.50 (108.54)**	**–2.62 (28.70)**	**1.56 (6.16)**	**10.59 (24.79)**
AUC_CAR_	–	−0.9	0.29	0.10
AUC_ANTE_		–	−0.34^*^	−0.21
AUC_INDUCTION_			–	0.001
AUC_POST_				–

CAR, cortisol awakening response; AUC, area under the curve; AUC_ANTE_, AUC before stress induction; AUC_INDUCTION_, AUC during stress induction; AUC_POST_, AUC after stress induction.

* *p < 0.05*.

### Associations between cortisol levels and cognitive performance

Table 2 reports the correlation coefficients between cortisol levels (AUCs) and cognitive performance.

**Table 2:** Correlation coefficients between cortisol levels (AUCs) and cognitive performance (delayed/immediate verbal/figural learning).

	**Immediate verbal learning**	**Delayed verbal learning**	**Immediate figural learning**	**Delayed figural learning**
	**M (SD)**	**M (SD)**	**M (SD)**	**M (SD)**
	**10.08 (1.09)**	**7.85 (1.45)**	**18.33 (5.35)**	**17.56 (5.31)**
AUC_CAR_	−0.26	−0.001	−0.22	−0.26
AUC_ANTE_	−0.32^*^	0.17	0.09	0.10
AUC_INDUCTION_	−0.28	−0.45^**^	0.07	−0.30
AUC_POST_	0.18	−0.09	−0.29	0.01

CAR, cortisol awakening response; AUC, area under the curve; AUC_ANTE_, AUC before stress induction; AUC_INDUCTION_, AUC during stress induction; AUC_POST_, AUC after stress induction.

* p < 0.05;

** *p < 0.01*.

Baseline cortisol levels (AUC_CAR_) were not statistically significantly associated with cognitive performance (verbal and figural encoding, storage and retrieval; all rs < 0.04, ps > 0.45).

Higher cortisol levels during memory encoding (AUC_ANTE_) were associated with lower immediate free recall performance for verbal (*r* = −0.32, *p* < 0.05), but not for figural performance.

A lower memory encoding performance (immediate free recall for verbal, but not for figural encoding) was associated with higher cortisol levels during memory storage (AUC_INDUCTION_; *r* = −0.18, *p* < 0.1) and during memory retrieval (AUC_POST_; *r* = −28, *p* < 0.05).

A higher cortisol level during memory storage (AUC_INDUCTION_) was associated with lower delayed cognitive verbal (*r* = −0.45, *p* < 0.01), and figural (*r* = −0.31, *p* < 0.05) performance.

Cortisol levels during delayed memory retrieval (AUC_POST_) were not associated with cognitive verbal and figural performance (encoding, storage, retrieval).

### Partial correlations between AUC_INDUCTION_ and delayed cognitive performance (controlling for cognitive performance during immediate recall)

A higher cortisol level during memory storage (AUC_INDUCTION_) was associated with lower delayed cognitive verbal (*r* = −0.45, *p* < 0.01), and figural (*r* = −0.31, *p* < 0.05) performance. When the cognitive performance during immediate recall (memory encoding) was introduced as co-variate, correlation coefficients collapsed from *r* = −0.45 and *r* = −0.31 to *r* = 0.08 and *r* = −0.07, suggesting that cognitive performance during encoding, but not cortisol secretion under stress conditions (AUC_INDUCTION_) predicted cognitive performance during delayed recall.

### Sleep parameters, cortisol levels and cognitive performance

Table 3 shows the descriptive statistical indices of objective sleep parameters and correlation coefficients between sleep parameters, cortisol levels and cognitive performance.

**Table 3:** Correlation coefficients between sleep parameters, cortisol levels (AUCs) and cognitive performance (immediate/delayed × verbal/figural learning).

	**Total sleep time**	**Sleep period time**	**Sleep efficiency**	**Sleep onset latency**	**Awakenings**	**Time awake**
**M (SD)**	**541.15 (39.45) min**	**540.41 (41.80) min**	**99.51 (0.74) %**	**10.21 (3.64) min**	**2.53 (3.12) nr**	**3.68 (5.35) min**
AUC_CAR_	0.02	−0.124	−0.23	−0.23	−0.12	0.03
AUC_ANTE_	−0.19	0.46^**^	0.17	−0.06	−0.23	−0.08
AUC_INDUCTION_	−0.7	−0.38^*^	−0.16	0.45^**^	−0.30	0.35^*^
AUC_POST_	0.05	−0.29	−0.05	0.19	0.14	−0.20
Immediate verbal learning	−0.10	−0.20	−0.12	0.03	0.05	0.01
Delayed verbal learning	−0.39^*^	0.13	−0.15	−0.36^*^	0.17	−0.23
Immediate figural learning	0.02	0.13	−0.12	−0.15	0.40^*^	0.07
Delayed figural learning	0.03	0.13	−0.12	−0.14	0.37^*^	0.05
	**Stage 1**	**Stage 2**	**Stage 3**	**Stage 4**	**Slow wave sleep**	**REM sleep**	**REM latency**	**REM density**
**M (SD)**	**4.60 (3.98) min**	**232.74 (55.38) min**	**47.41 (16.70) min**	**109.94 (15.2) min**	**159.17 (23.29) min**	**115.74 (30.93) min**	**90.35 (31.37) min**	**3.97 (1.06) min**
AUC_BASELINE_	0.16	−0.01	0.16	−0.29	0.61^**^	−0.03	−0.19	−0.13
AUC_ANTE_	0.23	0.21	0.08	−0.02	0.04	−0.03	−0.06	−0.06
AUC_INDUCTION_	−0.10	−0.27	0.32^*^	0.33^*^	0.20	0.05	0.06	0.10
AUC_POST_	−0.12	−0.22	0.15	0.19	−0.03	−0.21	−0.21	0.06
Immediate verbal learning	0.15	−0.01	−0.35^*^	0.02	−0.38^*^	0.03	−0.01	0.04
Delayed verbal learning	0.50^**^	0.14	−0.37^*^	−0.28	−0.11	−0.03	−0.04	−0.09
Immediate figural learning	−0.20	0.03	−0.26	−0.01	0.01	−0.29	−0.31	−0.18
Delayed figural learning	−0.15	0.08	−0.25	−0.01	−0.02	−0.29	−0.26	−0.22

* p < 0.05;

** *p < 0.01*.

With some few exceptions (see Table 3) the general pattern showed no significant associations between sleep patterns, cortisol secretion, and cognitive performance.

## Discussion

The key findings of the present study were that among a sample of 9-years old children a higher cortisol secretion was associated with lower cognitive performance during encoding. Further, cognitive performance before and after a stress challenge (Trier Social Stress Test for Children; TSST-C) decreased, though decrease was not affected by cortisol levels during the stress challenge (AUC_INDUCTION_). Last, objective sleep patterns and both cortisol under baseline (CAR) and challenge conditions (TSST-C) were unrelated. The pattern of results adds to the current literature in an important way, as it revealed a direction of influence neglected so far: The cognitive performance also impacted on neuroendocrinological processes (here: cortisol secretion), while previous studies exclusively focused on the influence of neuroendocrinological processes such as cortisol secretion under challenge conditions on cognitive information elaboration. Thus, in our opinion, we filled a gap in the scientific field, in that we were able to show the bidirectional association between cognitive and neuroendocrinological processes.

More specifically, we hold that with the present study we expanded upon the current literature in the following important ways. First, we performed this study with 9-years old children, and following Hollanders et al. (2017) research on stress-axis reactivity on social stress was mainly performed with 8-years old, or 10- to 16-years old children and adolescents. Second, to the best of our knowledge, no study has investigated the influence of the TSST-C on figural memory performance. Accordingly, we broadened the variety of cognitive outcome variables. Third, similarly, while previous studies mainly focused on the endocrinological response to a social stress condition, we were also interested in assessing the cognitive consequences of such an intervention. Fourth, we were also interested in investigating, if and if so, to what extent the cognitive performance might have had an influence on the stress axis activity, thus reversing the whole paradigm of cause and consequences. As a result, for instance in clinical settings, the bidirectional association between cognitive performance and stress axis activity should be considered as a possible confounder. Accordingly, and to a larger extent, when applying both the TSST-C and the TSST in school settings, clinical settings, and research settings, cognitive processes preceding the stress induction should be carefully monitored. In this view, in a previous study with children with separation anxiety disorder (Brand et al., 2011), we showed that anticipating separation and a social challenge led to higher cortisol secretions already before the stress challenge.

Four hypotheses and one research question were formulated, and each of these is considered now in turn.

With the first hypothesis, we assumed that a higher cortisol secretion would impair verbal working memory encoding, and data did confirm this. Accordingly, the present findings are in accord with a host of previous research (Sauro et al., 2003; Het et al., 2005; Quesada et al., 2012; de Veld et al., 2014; Shields et al., 2015, 2016, 2017; Starcke and Brand, 2016). However, we expand upon previous research in two ways: We showed that higher cortisol levels impacted on working memory encoding among about 9-years old children, while the only two available studies in about 9–10 years old children (Quesada et al., 2012; de Veld et al., 2014) investigated the influence of cortisol levels on long-term memory retrieval.

With the second hypothesis, we assumed that a psychosocial stressor (TSST-C) impaired retrieval of verbal working memory, but data did not confirm this. Accordingly, the present findings are at odds with a host of previous research (Sauro et al., 2003; Het et al., 2005; Quesada et al., 2012; de Veld et al., 2014; Shields et al., 2015, 2016, 2017; Starcke and Brand, 2016). To explain this gap, we note the particular study design of the present study, in that also the cognitive performance before the stress induction was taken into account (AUC_INDUCTION_ and delayed verbal recall: without confounder: *r* = −0.45; with confounder: *r* = 0.08). Likewise, a deeper introspection of the publications of (Quesada et al., 2012; de Veld et al., 2014) showed, that in these studies, cognitive performances during encoding were statistically not taken into account.

With the third hypothesis, we expected that poor sleep and increased morning cortisol secretion were associated, however, again, data did not support this. Accordingly, the present data are at odds with previous findings (Hatzinger et al., 2008, 2010, 2012; Hori et al., 2011; van Dalfsen and Markus, 2018). To explain this gap between previous research and the present data, we speculate that either the sample size was too low, thus, for these variables, the study was underpowered, or that the sample itself was biased: actually, from the sleep parameters it appears that children's sleep was quite stable (sleep efficiency: 99.5%; sleep onset latency: 10.21 min) and quantitatively and qualitatively high, such to impede statistical variance and therefore the odds of a detecting significant associations with other dimensions.

With the fourth and last hypothesis, we assumed that poor sleep, and high cortisol level under baseline (CAR) and under challenge conditions (TSS-C) would be associated, however, data did not fully confirm this, and again, the present findings are in contrast with previous studies (Hatzinger et al., 2007; Lemola et al., 2015; Perkinson-Gloor et al., 2015; Maurer et al., 2016). However, we also note that those children with higher baseline cortisol levels (AUC_CAR_) also displayed higher cortisol levels under challenge conditions (TSST-C: AUC_BASAL_), suggesting that in those children, a generalized over-active HPA-A might be assumed (Hatzinger et al., 2010, 2012, 2013a). In our opinion, the latter observation is also in accord with those studies showing an association between the increased cortisol secretion, the saturation of glucocorticoid-receptors, a negative feedback loop and a decreased cognitive performance (Harris et al., 2013; Finsterwald and Alberini, 2014).

Our final research question examined if and to what extent retrieval of working memory performance of figural information (Rey Osterrieth Complex Figure Test) would be influenced by cortisol levels as a proxy of psychosocial stress (TSST-C). The pattern of results was identical to the results of the verbal working memory task, or simply put: The performance on the figural working memory task before the psychosocial stress predicted the performance on the figural working memory task after the psychosocial stress, while again, the cortisol secretion did not confound this result (that is: stress-related cortisol levels did not influence the delayed figural working memory performance). Further, as shown in Table 3, figural working and long-term memory performance were virtually not associated with cortisol levels, suggesting that information processing of figural information might not rely on those neuronal pathway affected by the HPA-AA.

Despite the new findings, several limitations warn against an overgeneralization of the present results. First, the sample might be considered rather small, and accordingly, a larger sample might have yielded further statistically significant associations. Second, we tested explicit memory performances, whereas the broad range of implicit learning such as skill acquisition, priming, conditioning and non-associative learning (habituation, sensitization) were not assessed. Likewise, it remained unclear if participants with higher cognitive performances used techniques to improving memory such as chunking, imagery, or rehearsal during the learning phase to improve retrieval. Third, it is conceivable that meta-cognitive processes (“what do I think about my thinking?”) and emotional and motivational processes accompanying the learning phase of encoding, storage, and retrieval might have biased two or more dimensions in the same or opposite directions. Fourth, while the Yerkes-Dodson law postulates non-linear associations between neurophysiological arousal and cognitive performance, we did fully rely on linear associations. Fifth, given that 9-years old children are asked to perform and present their efforts in front of their class mates, it is conceivable that the task per se was perceived as too “harmless” and trivial to boost the HPA axis: Actually, performing in front of class mates is part of a young students' task of everyday school life.

## Conclusions

While a higher explicit cognitive performance (encoding) leads to a decreased cortisol secretion, the underlying cognitive-emotional and endocrinological processes remain unclear. It appears therefore that thorough and explicit learning reduces neurophysiological arousal; probably, knowing about one's own robust learning performance leads to reduced neurophysiological arousal and higher self-esteem. By constrast, emotions such as anxiety and insecurity might impede successful explicit learning. Further, against expectations, nor the cortisol secretion under baseline and challenge conditions were associated with objective sleep dimensions and cognitive performance. Overall, the pattern of results suggests that the association between cognition, sleep and cortisol secretion appears to be more complex, at least among 9-years old children, and among the present specific sample.

## Ethics statement

This study was carried out in accordance with the rules laid down in the Declaration of Helsinki and its later amendments. The protocol was approved by the local ethics committee of Basel, Switzerland.

## Author contributions

SB, TM, NK, DSB, SaL, MG, SeL, MB, UP, EH-T, and MH: study design, interpretation of the data, writing the draft, and final manuscript. SB, NK, MB, and MH: data gathering. SB, TM, NK, DSB, SaL, MG, SeL, MB, and MH: data analysis. SB, TM, NK, DSB, and MB: integration of the authors' comments.

### Conflict of interest statement

The authors declare that the research was conducted in the absence of any commercial or financial relationships that could be construed as a potential conflict of interest.
